# Socioeconomic factors and financial burdens of research “gap years” for dermatology residency applicants

**DOI:** 10.1097/JW9.0000000000000099

**Published:** 2023-08-04

**Authors:** Audrey Jacobsen, Gowri Kabbur, Rebecca L. Freese, Katelyn J. Rypka, Noah Goldfarb

**Affiliations:** a Department of Dermatology, Veterans Affairs Medical Center, Minneapolis, Minnesota; b Department of Dermatology, Hennepin County Medical Center, Minneapolis, Minnesota; c Department of Dermatology, Cleveland Clinic, Cleveland, Ohio; d Clinical and Translational Science Institute, Biostatistical Design and Analysis Center, University of Minnesota, Minneapolis, Minnesota; e Department of Dermatology, University of Minnesota, Minneapolis, Minnesota; f Department of Internal Medicine, Veterans Affairs Medical Center, Minneapolis, Minnesota

**Keywords:** dermatology, gap year, graduate medical education, match, research year, residency

**What is known about this subject in regard to women and their families?**

- There is a paucity of available literature regarding the burdens of “gap years” specific to female dermatology residency applicants.

**What is new from this article as messages for women and their families?**

- Our study indicates a potential gender bias in the match application process. We found that female dermatology residents, when controlling for marital status, showed a higher tendency to take a gap year compared to male residents. However, caution is required when interpreting this finding due to the small sample size, limited response rate, and potential recall bias in our survey study.

A trend among dermatology residency applicants is pursuing a “gap year” dedicated to research or obtaining an additional degree to strengthen their chances of matching into this competitive specialty.^1,2^ This training interruption does not include the $11,324 that the average matched dermatology applicant spent on applications, away rotations, and interviews before the COVID-19 pandemic.^3^ We aimed to assess demographics, financial burden, and monetary support of dermatology residents who utilized a gap year.

An online questionnaire was distributed, November 2019 to January 2020, to dermatology program directors to share with current residents at Accreditation Council for Graduate Medical Education approved programs in the United States and Canada. Data were analyzed in STATA. Institutional Review Board exempt-STUDY00007177 consent was waived.

Of the 181 respondents (estimated 14.5% response rate of total dermatology residents enrolled in ACGME programs), 65.9% (*n* = 122) were female and 67.4% (*n* = 120) self- identified as White, while 20.02%, 2.2%, and 10.1% identified as Asian, Black, and other/multiple, respectively. A third (*n* = 60) reported undergoing a “gap year.” Single students were more likely to take a gap year than their married counterparts (*P* = .041). A logistic regression analysis, controlling for marital status, demonstrated that females had 2.32 times increased odds of taking a gap year (95% CI [1.15–4.87]; *P* = .02) compared to males (Table 1). Of gap-year takers, 71.7% reported some concurrent financial burden; 40.0% reported using personal savings to fund much of their research year; 13.3% utilized additional loans and 46.7% received a grant, or faculty/institutional stipend. Of grants/stipend recipients, the average monthly funding was $2,470.40 (Table 2).

**Table 1:** Demographics, loan burden, and USMLE scores of gap year versus nongap year respondents

	All respondents	No gap year respondents	Gap year respondents	*P*-value^a^
Overall	188	121 (66.9%)	60 (33.1%)	–
Age, mean (SD)	30.0 (2.48)	30.2 (0.32)	29.9 (0.23)	.466
Gender, *n* (%)				
Female	122 (65.9)	73 (60.3)	45 (76.3)	.052
Male	63 (34.1)	48 (39.7)	14 (23.7)	
Marital status, *n* (%)				
Single	78 (42.6)	43 (36.1)	32 (53.3)	.041
Married	105 (57.4)	76 (63.9)	(46.7)	
Race, *n* (%)^b^				
Asian	36 (20.2)	21 (17.9)	15 (26.3)	.323
Black	4 (2.2)	2 (1.7)	2 (3.5)	
Other/multiple	18 (10.1)	11 (9.4)	7 (12.3)	
White	120 (67.4)	83 (70.9)	33 (57.9)	
Self-identified family income as child, *n* (%)				
Low-income	7 (3.8)	6 (5.1)	1 (1.7)	.418
Lower-middle class	60 (33.0)	38 (32.2)	22 (36.7)	
Upper-middle class	95 (52.2)	63 (53.4)	28 (46.7)	
Upper class	20 (11.0)	11 (9.3)	9 (15.0)	
Medical school loans, *n* (%)				
No	57 (31.3)	35 (28.9)	22 (36.7)	.376
Yes	125 (68.7)	86 (71.1)	38 (63.3)	
Medical school loan burden ($), *n* (%)				
$0–49,999	7 (5.6)	5 (5.8)	2 (5.3)	.865
$50,000–99,999	16 (12.8)	12 (14.0)	4 (10.5)	
$100,000–149,999	15 (12.0)	12 (14.0)	3 (7.9)	
$150,000–199,999	31 (24.8)	21 (24.4)	10 (26.3)	
$≥200,000	56 (44.8)	36 (41.9)	19 (50.0)	
Step 1 score, *n* (%)				
<240	37 (20.4)	21 (17.5)	16 (26.7)	.215
≥241	144 (79.6)	99 (82.5)	44 (73.3)	

USMLE, United States Medical Licensing Examination.

a Pearson χ^2^ tests for measure of association were used with significance set at *P* ≤ .05.

b Hispanic, Latino/a, or Spanish origin was also included as ethnicity option in the original survey, with 6 (54.6%) and 5 (45.5%) in the gap year and nongap year participants, respectively.

**Table 2:** Funding and financial burden for respondents taking a gap year

Questions for respondents who reported taking a gap year	Responses, *n* = 60
All sources of financial support for cost of living during gap year (not including direct research costs), one or more answers may be selected	*n* (%)
Personal savings or family	36 (60.0)
Stipend by faculty/research mentor	8 (13.3)
Outside grant personally obtained	6 (10.0)
Institutional stipend	24 (40.0)
Additional loans (federal or private)	11 (18.3)
Other	2 (3.3)
*Majority* source of financial support for cost of living during gap year (not including direct research costs)	*n* (%)
Personal savings or family	24 (40.0)
Stipend by faculty/research mentor	1 (1.7)
Outside grant personally obtained	6 (10.0)
Institutional stipend	21 (35.0)
Additional loans (federal or private)	8 (13.3)
Approximate monthly amount ($) if majority support from stipend, grant, or institutional support, mean (SD)	2,470.4 (953.06)
Would still have taken a research year without external funding?^a^	*n* (%)
Yes	15 (45.5)
No	18 (54.6)
If external funding was obtained, how well did this cover personal expenses?	*n* (%)
Completely covered expenses	19 (57.6)
Covered more than half of expenses	10 (30.3)
Covered less than half of expenses	3 (9.1)
Did not at all cover my expenses	1 (3.0)
Financial burden or hardship (if any) during research fellowship?	*n* (%)
No financial burden or hardship	17 (28.3)
Minimal financial burden or hardship	12 (20.0)
Some financial burden or hardship	16 (26.7)
Moderate financial burden or hardship	7 (11.7)
High financial burden or hardship	8 (13.3)

a External funding is defined as funding from a stipend supported by a faculty/research mentor, grand personally obtained, or an institutional stipend.

Nearly 80% of gap-year takers utilized personal savings, family support, and/or federal or private loans to help cover expenses, with a high rate of financial burden reported. For those who secured funding, the average amount was under 250% of the federal poverty level, raising the ethical question of whether gap years should be encouraged, even when they are “funded.” Without monetary support, over half of the respondents reported they would not have taken a gap year, emphasizing the importance of funding.

Our study may suggest a potential gender bias as female residents were more likely than males to take a gap year when controlling for marital status in our study. However, this finding should be interpreted with caution given the small sample size. Other limitations to our study include a limited response rate and potential recall bias.

While our study did not demonstrate significant associations between gap years and race, ethnicity or socioeconomic backgrounds, dermatology remains one of the least diverse medical specialties.^2^ Others have raised concern that the pressure for a gap year may introduce systematic bias into the applicant pool^2^ especially when no significant difference in match rates has been reported following gap years, except for potentially matching into top-ranked programs.^4^

In conclusion, gap years create a significant financial burden for medical students hoping to match into dermatology. This pressure to complete a gap year may self-select for more affluent applicants. More studies are needed to evaluate the true impact this has on diversity within the specialty.

## Conflicts of interest

NG participated in clinical trials for Abbvie, Pfizer, Chemocentrix, and DeepX Health and has served on advisory boards and consulted for Novartis and Boehringer Ingelheim. The other authors have no conflicts of interest.

## Funding

AJ was supported by the National Institutes of Health’s National Center for Advancing Translational Sciences (grant UL1TR002494). GK was supported by the National Institutes of Health’s National Center for Advancing Translational Sciences (grant UL1TR002494).

The content is solely the responsibility of the author and does not represent the views of any affiliated organizations.

## Study approval

The authors confirm that any aspect of the work covered in this manuscript that has involved human patients has been conducted with the ethical approval of all relevant bodies.

## Author contributions

AJ and GK: Participated in research design, performance of research, data analysis, and writing of the paper. RLF: Participated in data analysis and writing of the paper. KJR: Participated in writing of the paper. NG: Participated in research design and writing of the paper.

## Acknowledgments

We gratefully acknowledge the contributions of Drs. Baho Sidiqi, MD, MPH, Erin F. Gillespie, MD, Chunyu Wang, MD, Melissa Dawson, MS, and Abraham J. Wu, MD, in their excellent article titled “Mind the Gap: An Analysis of ‘Gap Year’ Prevalence, Productivity, and Perspectives Among Radiation Oncology Residency Applicants,” which provided a framework for the survey tool utilized in our study, with authors’ permission.
